# Supplementary material for: Tumor Size Matters—Understanding Concomitant Tumor Immunity in the Context of Hypofractionated Radiotherapy with Immunotherapy
Source: Cancers (Basel). 2020 Mar 18;12(3):714. doi: 10.3390/cancers12030714 (PMC7140082; doi:10.3390/cancers12030714)
Supplement: Supplementary file 1 [file cancers-12-00714-s001.pdf]

Supplementary Materials

Tumor Size Matters—Understanding Concomitant Tumor Immunity in the Context of Hypofractionated Radiotherapy with Immunotherapy

Jean Philippe Nesseler, Mi-Heon Lee, Christine Nguyen, Anusha Kalbasi, James W. Sayre, Tahmineh Romero, Philippe Nickers, William H. McBride and Dörthe Schaue

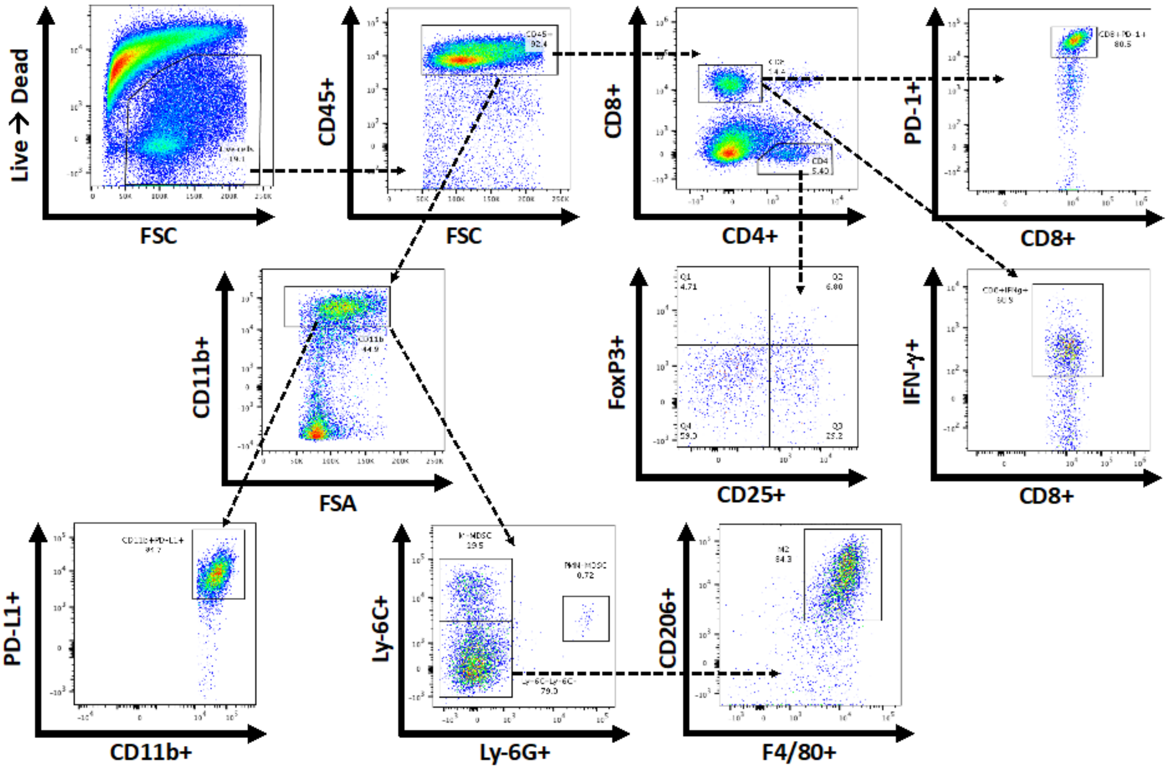

Figure S1. Multi-parametric flow cytometry gating strategies for lymphoid and myeloid cell populations.

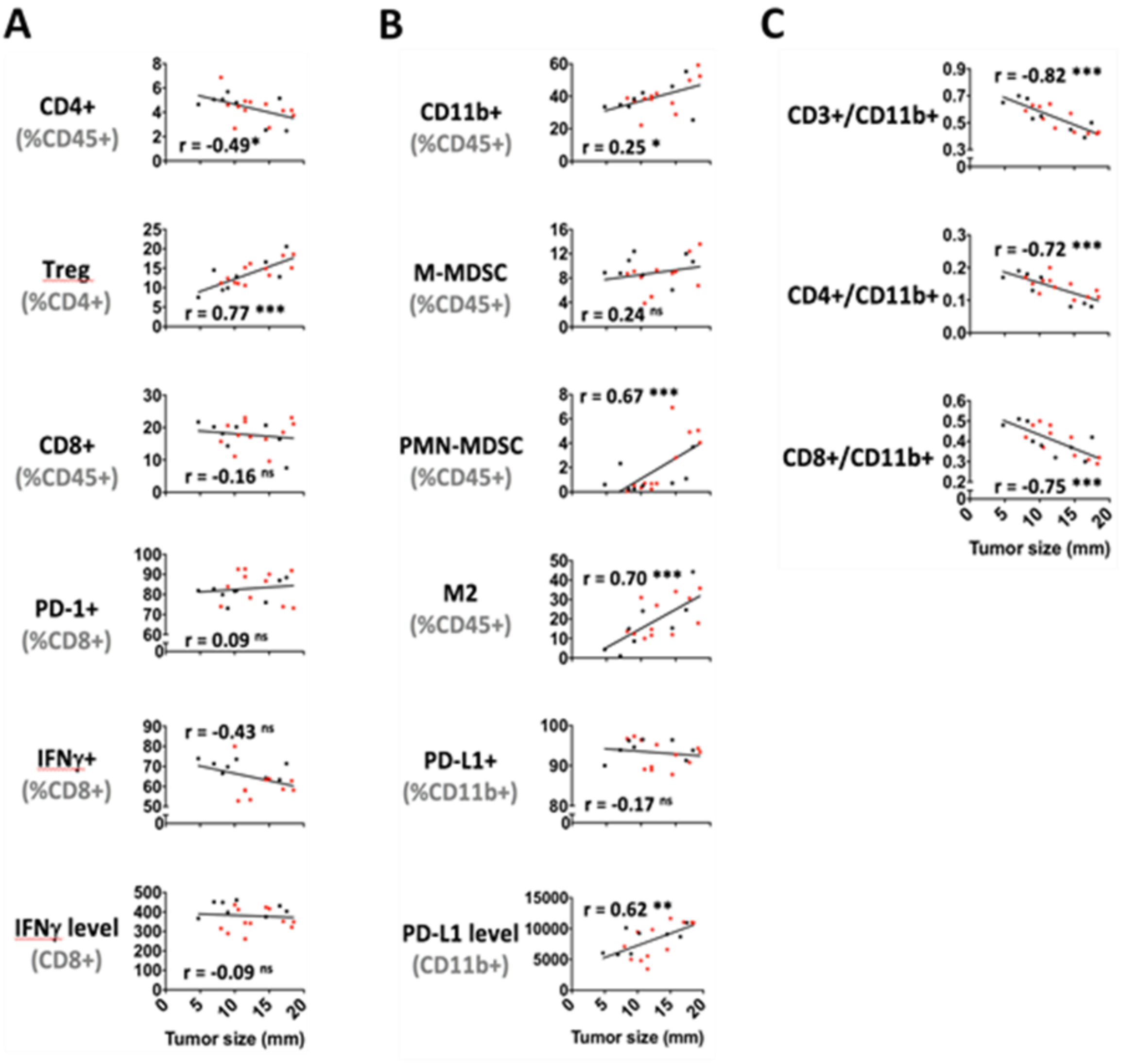

Figure S2. The immune cell profile in primary tumors (T1) depends on their tumor size. There is no obvious relation with the development of a secondary tumor T2. (**A**) Lymphoid subsets; (**B**) Myeloid subsets; (**C**) Ratio of lymphoid-to-myeloid subsets. (n = 20). Black dots are data from T1 tumors in mice with palpable T2 tumors, red dots represent mice in which T2 did not grow.

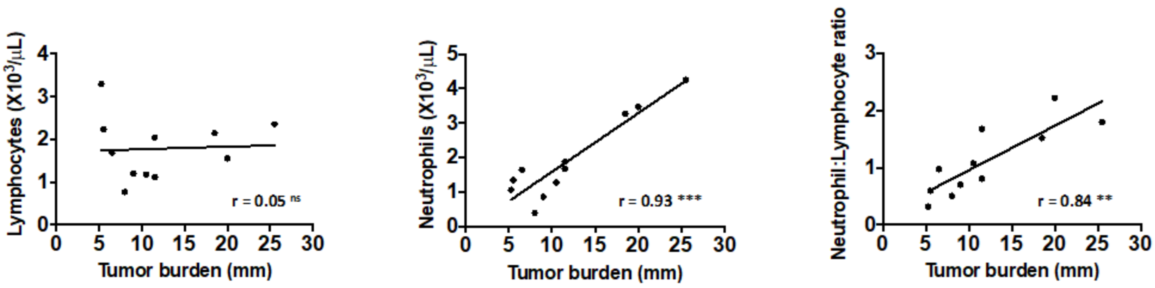

Figure S3. Linear regression analysis for circulating lymphocyte counts (left), neutrophil counts (middle), and the neutrophil/lymphocyte ratio (right) versus tumor burden. ** $p < 0.01$; *** $p < 0.001$; ns = not significant.

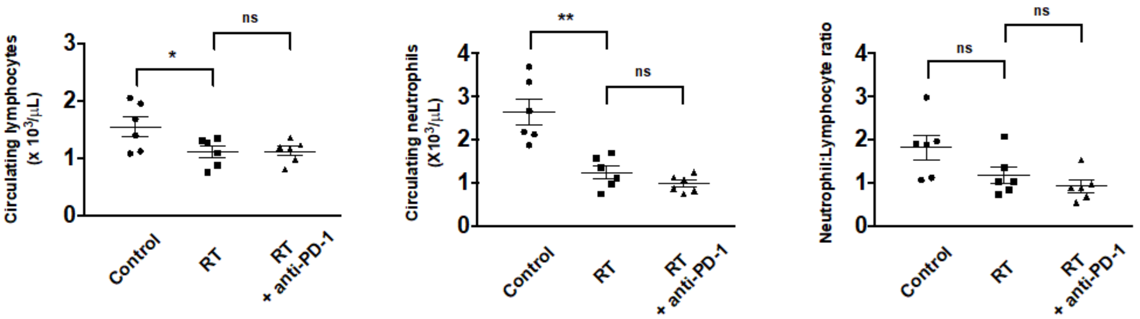

Figure S4. Impact of radiation treatment alone or in combination with PD-1 blockade on circulating lymphocyte (left) and neutrophil counts (middle), and on the ratio of neutrophils/lymphocytes (right) at d18 (n = 6 per treatment group). Data are mean +/− SEM. * $p < 0.05$; ** $p < 0.01$; ns = not significant.

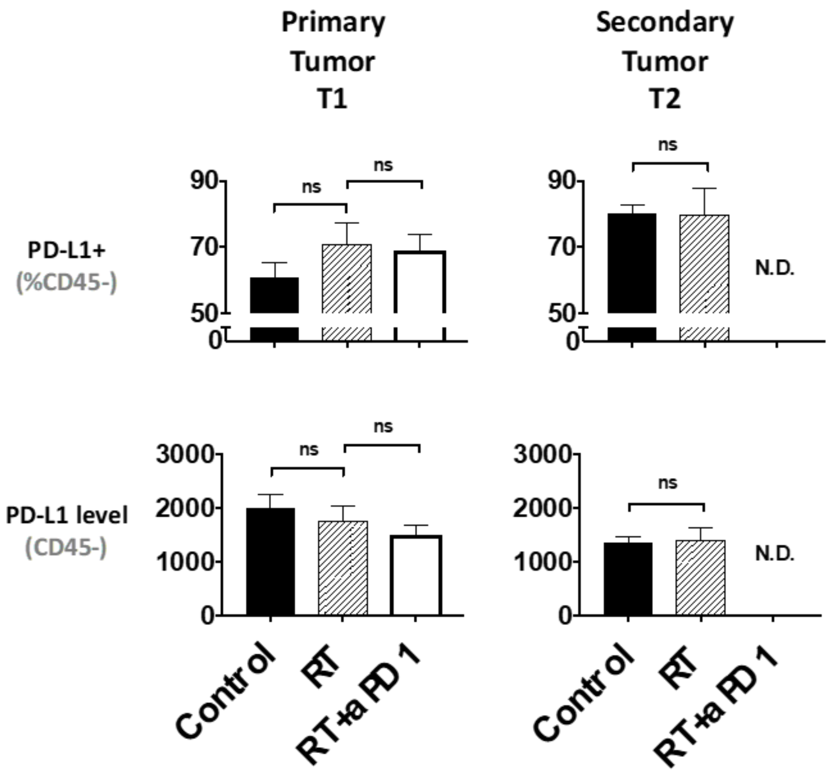

Figure S5. PD-L1 expression on tumor cells defined as CD45$^-$ in the primary tumor T1 and the secondary tumor T2 in response to radiation therapy (RT) with or without anti-PD-1 (RT + aPD1) (n = 6 per group). Data are mean +/− S.E.M. with ns not significant; ND: No Data.

Table S1. Phenotypic markers to define immune cells.

Cell Type	Markers
CD8 T cell	CD45+ CD8+ CD4-
CD4 T cell	CD45+ CD8- CD4+
Regulatory T cell	CD45+ CD8- CD4+ CD25+ FoxP3+
M-MDSC	CD45+ CD11b+ Ly6G- Ly6C+
PMN-MDSC	CD45+ CD11b+ Ly6G+ Ly6C(int)
M2 macrophages	CD45+ CD11b+ Ly6G- Ly6C- F4/80+ CD206+

© 2020 by the authors. Licensee MDPI, Basel, Switzerland. This article is an open access article distributed under the terms and conditions of the Creative Commons Attribution (CC BY) license (http://creativecommons.org/licenses/by/4.0/).
